# Practitioner’s review: medication for children and adolescents with autism spectrum disorder (ASD) and comorbid conditions

**DOI:** 10.1007/s40211-021-00395-9

**Published:** 2021-06-23

**Authors:** Christian Popow, Susanne Ohmann, Paul Plener

**Affiliations:** Dept. Child and Adolescent Psychiatry, Medical University of Vienna, Waehringer Guertel 18–20, 1090 Vienna, Austria

**Keywords:** Autism spectrum disorder, ADHD, Children and adolescents, Pharmacotherapy, Autismus-Spektrum-Störung, ADHS, Kinder und Jugendliche, Pharmakotherapie

## Abstract

Alleviating the multiple problems of children with autism spectrum disorder (ASD) and its comorbid conditions presents major challenges for the affected children, parents, and therapists. Because of a complex psychopathology, structured therapy and parent training are not always sufficient, especially for those patients with intellectual disability (ID) and multiple comorbidities. Moreover, structured therapy is not available for a large number of patients, and pharmacological support is often needed, especially in those children with additional attention deficit/hyperactivity and oppositional defiant, conduct, and sleep disorders.

## Abstract (de)

Die Linderung der vielfältigen Probleme von Kindern mit Autismus-Spektrum-Störung (ASS) und ihrer Begleiterkrankungen stellt für die betroffenen Kinder, Eltern und Therapeuten eine große Herausforderung dar. Aufgrund einer komplexen Psychopathologie reichen strukturierte Therapie und Elterntraining nicht immer aus, insbesondere bei Menschen mit geistiger Behinderung (GB) und multiplen Komorbiditäten. Darüber hinaus steht für viele Patienten keine strukturierte Therapie zur Verfügung, und häufig ist pharmakologische Unterstützung erforderlich, insbesondere bei Kindern, bei denen eine Aufmerksamkeitsdefizit‑/Hyperaktivitätsstörung und oppositionelle Trotz‑, Verhaltens- oder Schlafstörungen hinzukommen.

## Introduction

Autism spectrum disorder (ASD) is a common [73], complex, genetically based, disabling disorder [15] that needs specific knowledge and parenting skills [165] and burdensome, costly treatment. The complex clinical picture is characterized in ICD-11 6A02 [320] by

- Persistent deficits in the ability to initiate and sustain reciprocal social interaction and social communication,
- A range of restricted, repetitive, and inflexible patterns of behavior and interests, and
- A high prevalence of intellectual disability, language impairments, and other comorbid disorders

and a number of comorbid conditions such as attention deficit/hyperactivity disorder (ADHD), sleep disorders, convulsions, oppositional defiant disorder (ODD), anxieties, obsessions and compulsions (OCD), depression, and numerous other symptoms and conditions that are discussed as to whether they represent “core” or comorbid problems [281]. These conditions differ in symptomatology, prevalence, and treatability from those of normally developing children. These differences, partly related to the reduced flexibility (for change), partly to genetic and social conditions, may render therapy and its prognosis difficult, and will increase the impairments of self-worth/self-efficacy and the tendency for depression in the children on the spectrum. Comorbid conditions also seem to contribute to the increased mortality of children with ASD [304].

**Table 1:** Abbreviations

Abbrev.	Definition	Abbrev.	Definition
ABA	Applied behavioral analysis	IQ	Intelligence (Quotient)
ACTH	Adrenocorticotropic hormone, corticotropin	LGS	Lennox–Gastaut syndrome
AD	Antidepressant	LKS	Landau–Kleffner syndrome
AD		MAOI	Monoamino oxidase inhibitor
ADHD	Attention deficit/hyperactivity syndrome	MPEP	2‑methyl-6- (phenylethynyl)pyridine
BD	Bipolar disorder	MT1	Melatonin 1 (receptor)
ASD	Autism spectrum disorder	NDRI	Norepinephrine-dopamine reuptake inhibitor
BPD	Borderline personality disorder	NMDA	N‑methyl-D-aspartate
CBT	Cognitive behavioral therapy	OCD	Obsessive compulsive disorder
CSWS	Continuous spike waves during slow-wave sleep	ODD/CD	Oppositional defiant disorder/conduct disorder
DSM‑5	Diagnostic and Statistic Manual for Mental Disorders, 5th edition	PE	Partial epilepsy
DRESS	Drug rash with eosinophilia and systemic symptoms	PECS	Picture exchange communication system
EF	Executive functions (functioning)	REM sleep	Rapid eye movement sleep
ESES	Electrical status epilepticus during slow-wave sleep	RLS	Restless legs syndrome
FDA	Food and Drug Administration	SGA	Second generation antipsychotic
FGA	First generation antipsychotic	SSRI	Selective serotonin reuptake inhibitor
FXS	Fragile X syndrome	SNRI	Selective serotonin and norepinephrine reuptake inhibitor
GABA	Gamma-amino-butyric acid	SE	Side effects
GAD	Generalized anxiety disorder	$t$ $1/2$	Half life
CBT	Cognitive behavioral therapy	TCA	Tricyclic antidepressant
ICD	International Classification of Diseases	TCM	Traditional Chinese medicine
ID	Intellectual disability	TEACCH	Treatment and education of autistic and related communication handicapped children
IGF‑1	Insulin-like growth factor – 1	VPS	Valproic acid

ASD comprises persons with a very low functional level up to a normal or even supranormal level with relatively low impairment. The disorder may not be cured but largely ameliorated by therapy and guided intrafamilial support [36, 165]. Especially in children with a low functional level, structured behavioral therapies [178] such as ABA^1^ and its variants, TEACCH^2^ or PECS^3^ have been proven to be beneficial. Therapeutic success will depend on the level of impairment, the intrafamilial and peer relation support, the availability, quality and quantity of therapeutic support [183, 192], the age at diagnosis [86, 119, 229, 263, 299], the types and number of comorbid conditions, and the financial support provided by the state or the social insurance, because an individual family will usually not dispose of the necessary means. Less affected children will present with flexibility problems and may easily be overburdened with social problems [166]. Additional challenges may be caused by comorbid conditions like ADHD, dysexecutive problems, depression, anxiety disorders, or seizures [10, 18, 24, 38, 105, 106, 187, 201, 281] (Table 2 [187, 223, 281]). Therapy should aim at attaining autonomy, flexibility, social competence, an educational level that is appropriate to the individual intellectual capacity of the child, and provide the basis for a self-determined and socially integrated life.

**Table 2:** ASD: relevant comorbid disorders

Disorders	Normotypic Children %	ASD Children %	References
Anxiety disorders	20–40	11–84	[281]
Sensory integration/EF	7.5–15	$> 75$	[126, 198]
Sleep disorder	22–32	40–80	[175]
ADHD	5–7	30–75	[10, 58, 266]
ODD/CD		30–90	[264]
Intellectual disability	2–3	25–70	[163]
OCD	2.5	8–37	[187]
Epilepsy	1–3	20–34	[24, 105, 261]
Depression/BPD	2–3	11–20	[161, 201]
Tic disorder	1–2	9–20	[260]
Central auditory processing disorder	2–5	?	[16]

“Conventional” pharmacotherapy is targeted to reduce inappropriate behavior and the associated burden for family, school, and the social environment, to limit inattention, impulsivity, and hyperactivity associated with ADHD, and to reduce the risk of seizures. Up to two-thirds of children with ASD are treated with psychotropics, and a third with multiple drugs [92, 156, 288]. Newer trends aim at improving social communication [21] or at transferring experimental therapies into real life [81, 171]. Examples include improving the imbalance between excitatory (glutamatergic) and inhibitory (GABA-ergic) neurotransmission [180, 216] or synaptic plasticity [34]. Among the most promising candidate substances are [171], NMDA^4^ antagonists [33], memantine [139], and d‑Cycloserine [68, 214], the GABA agonists, baclofen or arbaclofen [77, 130], oxytocin [17, 21, 47, 113, 313], vasopressin [235] or balovaptan [27], and insulin-like growth factors (IGF-I) [44, 301]. Among these, only the binding hormone oxytocin has gained widespread attention, stimulating a considerable number of clinical studies, although with inconsistent results [228].

In order to improve the multiple medical, social, behavioral, learning, or sleep-related problems, a number of drugs have been recommended and studied in clinical trials [241]. In addition, a number of experimental therapies, such as diets and brain extracts, were tried, most of them without any clinical evidence. Because the individual reaction to pharmacotherapy varies considerably [28], individualized treatment is mandatory [218]. We, therefore, performed a systematic review of the current literature, aiming at providing an overview on recommended pharmacotherapy for ASD and its most important comorbid disorders. The review is divided into three sections:

1. Pharmacologic agents
2. Therapy for common problems of ASD and comorbid disorders
3. Other substances, supplementary and alternative therapies.

## Methods

**Fig. 1:**
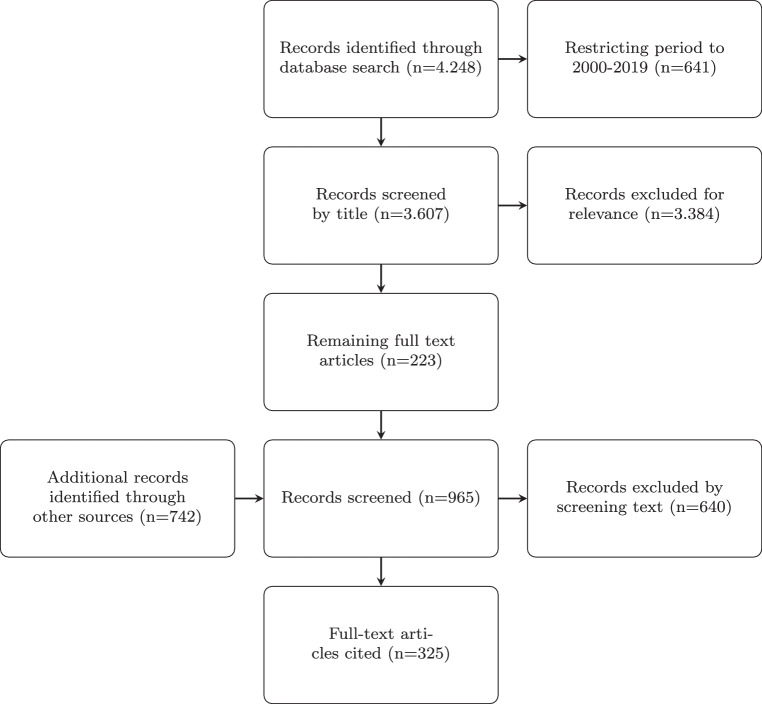
Processing of records

We searched the database PubMed/Medline for the following terms: autism AND pharmacotherapy OR medication, and retrieved 4.248 citations. Restricting the period covered to the years 2000–2019 and the language to English OR French OR German; 3.607 citations remained, including 1120 reviews. Selecting relevant titles, primarily taking into account the contents and quality of the papers, and secondarily the authors, publication media (impact factor), and date (selecting newer references), 223 remained. These were carefully studied in detail and supplemented by 742 additional relevant articles retrieved by specific topic searches that were considered important for understanding during the writing process. This added to 965 references of which 325 were cited in this article, depending on their subjectively estimated significance^5^, and aiming at not overloading the chapter with citations (see Fig. 1). The relationship between reviews and meta-analyses and original papers in the cited references was $1:3$.

## Pharmacotherapy of ASD

In the following, we will discuss the various groups of pharmaceuticals used in children and adolescents with ASD, namely antipsychotics, antidepressants, and anticonvulsants.

### Antipsychotics

Antipsychotics influence dopamine neurotransmission, act sedating in lower, antipsychotic in medium, and narcotic in high doses. First generation antipsychotics (FGA), especially haloperidol, have been shown to influence stereotypic and hyperactive behavior, to reduce temper tantrums and social isolation [9]. FGAs should no longer be used because of an inappropriate risk–benefit ratio related to cognitive as well as early and late (e.g., dyskinetic) side effects. As an alternative, second generation antipsychotics (SGAs), especially risperidone, aripiprazole, and quetiapine, are substances of choice for treating aggression, self-injuring behavior, temper tantrums, withdrawal, tics, and rituals.

This is also true for the SGA clozapine because of its dangerous hematologic side effects [152]. As an alternative, SGAs, especially risperidone, aripiprazole, and quetiapine, are substances of choice for treating aggression, self injuring behavior, temper tantrums, withdrawal, tics and rituals [35, 43, 62, 68, 103, 122, 153, 170, 221, 231, 241, 249, 262, 272, 290, 295, 319]. Other SGAs (such as asenapine and iloperidone) may also be used off-label but do not offer advantages [326]. Positive effects should be balanced against (metabolic, endocrine, neurologic, and cardiac) side effects [61, 273]. Therefore, mainly low-dose application should be tried. Recommended dosages and specific features are listed in Table 4. Adding topiramate to risperidone therapy was more effective on overall behavior when compared to risperidone monotherapy [257]. A potential adverse effect of topiramate on language development [227] has, nevertheless, to be considered.

**Table 3:** ASD Symptoms, comorbid disorders and (off-label) pharmacotherapy

Symptoms	Available drugs
Behavioral problems, restlessness, temper tantrums, self-injuring behavior	Antipsychotics, (anticonvulsants)
Social problems	Oxytocin, D‑cycloserin, memantine (experimental)
Sleeping problems	Melatonin, antipsychotics, antihistaminics
ADHD	Atomoxetin, methylphenidate, amphetamines, (guanfacine ER)
Tics	Antipsychotics, ($\alpha_{2}$ sympathomimetics, SSRIs)
Depression	SSRIs, SNRIs, (+ antipsychotics)
Bipolar disorder	Antipsychotics, (lithium)
Anxiety & OCD	SSRIs (higher dosage needed), pregabaline
Seizures	Valproic acid, levetiracetam, lamotrigine (and others)
Psychosis	Antipsychotics
GI problems	Diet? probiotics?

**Table 4:** Selected antipsychotics used in children and adolescents with ASD

Drug	$t$ $1/2$ ${}^{\text{a}}$	Recommended Dose (mg/kg/d)	Spec. remarks	References${}^{\text{b}}$
Risperidone	22 h${}^{\text{c}}$	0.005–0.02${}^{\text{d}}$ also available as syrup	Standard therapy${}^{\text{e}}$	[42, 64, 153, 207, 278]
Aripiprazole	60–80 h	0.05–0.1${}^{\text{f}}$	Standard therapy${}^{\text{g}}$	[46, 62, 66, 82, 196, 231]
Olanzapine	30–60 h	0.1	SE: sedation, metabolic	[93, 136, 291]
Paliperidone		0.5–2	No advantage over risperidone	[98]
Quetiapine	7 h	0.5–4	Also acts against GAD${}^{\text{h}}$	[109, 122, 200]
Ziprasidone	6 h	0.02–0.4	Cardiac SE (QTc $\uparrow$)	[69, 195]
Pimozide	55 h	0.02–0.08	FGA, therapy resistant tics	[79]

${}^{\text{a}}$ [110], ${}^{\text{b}}$ as related to ASD, ${}^{\text{c}}$ 9-hydroxyrisperidone, ${}^{\text{d}}$ also available as syrup ${}^{\text{e}}$ FDA approved from age 5 years on, ${}^{\text{f}}$ also available as solution, ${}^{\text{g}}$ FDA approved from age 6 years on, ${}^{\text{h}}$ GAD – generalized anxiety disorder

### Antidepressants

In normally developing children, selective serotonin antagonists (SSRIs) are effective against depressive symptoms with substance-related differences in effectivity and side effects. SSRIs also act against anxiety disorders in lower dosages and against OCD in higher dosages, compared to the treatment of depression. In children with ASD, SSRIs are widely prescribed, but their therapeutic effect is less evident [319]. Other AD agents, such as MAOIs, mirtazapine, hypericum, etc., also seem to produce only little effect, possibly because of elevated peripheral serotonin blood levels in a number of children and adolescents with ASD [100, 232, 309, 319].

A few studies suggest improvements of repetitive and stereotypic behavior with AD therapy in children with ASD [221], although this was not reported by King et al. [168] or Williams et al. [319]. Side effects of SSRIs usually are mild but may be exaggerated in children with ASD, especially when children are restless and agitated [173]. Bupropion, a NDRI^6^ acts like a stimulant, may create dependence, and should not be used in adolescents. Mirtazapine [243], a tricyclic AD, has modest antidepressant effects and further acts as a sedative and hypnotic agent by stimulating H1 receptors but is slowly eliminated ($t$ $1/2$ 37 h), strongly increases appetite, and leads to significant weight gain [143]. Studies in autistic children are scarce (e.g., [243]), and long-term studies are not available. Mirtazapine, therefore, should not be used or only used for a limited period and in low doses. Clomipramine and tricyclic antidepressants should only be used with care because of their severe side effects, and duloxetine and pregabaline have not been systematically studied in children and adolescents with ASD.

In summary, although AD medication, especially SSRIs, is widely prescribed in children and adolescents, its effectiveness is limited to not evident in children with ASD, and side effects may be more exaggerated in these patients. Therefore, the use of ADs in ASD can generally not be recommended. Because of their widespread use, pharmacologic data on AD medication are nevertheless summarized in Table 5.

**Table 5:** Selected antidepressants used in children and adolescents with ASD to treat depression, anxiety, and OCD

Drug	$t$ $1/2$ ${}^{\text{a}}$	Recommended Dose (mg/kg/d)	Specific remarks	Literature${}^{\text{b}}$
Fluoxetine	1–6 d	0.4–0.8	SE: sleep & eating problems	[135, 169, 253]
Paroxetine	12–22 h	0.4	Also effective against anxiety disord. and drug treatment	[242]
Sertraline	23–26 h	1	Well tolerated	[292]
Agomelatin	2.3 h	0.5–1	MT1 & $\beta 2$ agonist, no systematic studies in adolescents	[224]
Duloxetin	8–17 h	0.4–1.2	SNRI	[224]
Pregabalin	6 h	3–6–10	GABA analogon, pain killer, anticonvulsant, anxiolytic	No studies in ASD patients

${}^{\text{a}}$ [110], ${}^{\text{b}}$ as related to ASD

#### Anticonvulsants

Anticonvulsants may be used to treat epilepsies, bipolar disorders, and externalizing behavioral problems^7^. Anticonvulsant treatment of children with ASD [83, 133, 261], like in other patients with convulsions, depends on the type of convulsions and should always be combined with psychosocial support [261].

The most commonly used pharmacotherapeutics are valproic acid, lamotrigine, levetiracetam, and ethosuximide [96], cf. Table 6. In select syndromes such as Landau–Kleffner syndrome or ESES^8^, corticosteroids, ACTH, or immunoglobulin therapy may be considered [303]. Additional nonpharmacological therapeutic options for therapy-resistant epilepsies include vagus nerve stimulation [184], ketogenic diet, and neurosurgical interventions [114]. It is not clear whether an interictal epileptiform EEG may be a cofactor contributing to neurologic deterioration or progressing developmental retardation [310]. Pharmacologic treatment should always be considered if symptoms get worse.

**Table 6:** Anticonvulsants selected

Drug	$t$ $1/2$ (h)${}^{\text{a}}$	Recommended Dose (mg/kg/d)	Comments	References${}^{\text{b}}$
Ethosuximide	53	10–20–40	Absences, well tolerated	[95]e
			No effect on behavior, additive to VPS	
Valproic acid	12–16	10–15–30	Enhances GABA-ergic inhibition	[96, 136]
			Cortical hyperconnectivity, increases risk	
			Of ASD and malformation when	
			Administered during pregnancy	
Lamotrigine	25–50	0.5–4	Against gen. and PE, well tolerated	[23]
			Against BSD, no effect on behavior	
Levetiracetam	7	20–40–60	Against generalized and PE, SE tiredness	[96]
			No effect on behavior	
Clobazam	18	0.2–0.8	Add-on against prim. generalized and PE	[83]
Clonazepam	18–50	0.01–0.4	Against myoclonus epilepsy, SE: dizziness, ataxia	[83]
Gabapentin		10–40	Add-on against PE and sec. generalized	
			Epilepsy, SE tiredness, DRESS${}^{\text{c}}$	[115]
Sultiame	24	5–6	SE: ataxia, paresthesia, anorexia	
Topiramate	19–25	1–$4/2$	Against PE and generalized epilepsy,	
			LGS${}^{\text{d}}$, SE tiredness	
			Weight loss, cognitive	[68, 133]
			impairment	
Vigabatrin	5–8	20–$60/2$		

${}^{\text{a}}$ [110], ${}^{\text{b}}$ as related to ASD, ${}^{\text{c}}$ DRESS $=$ drug rash with eosinophilia and systemic symptoms, ${}^{\text{d}}$ LGS $=$ Lennox–Gastaut syndrome

### Therapy for Common Problems of ASD and Comorbid Disorders

Pharmacotherapy for patients with ASD aims at reducing inappropriate behavior and the related intrafamiliar and psychological stress, at improving engagement in therapy, health-related quality of life, performance at school and work, social integration and participation, and at treating comorbid problems such as ADHD or seizures [14, 53, 67, 72, 154, 156, 164, 180, 210, 220, 245, 274]. Limitations include inconsistent evidence of efficiency and side effects, especially with long-term use [107]. A recent study [53] compared the benefits and adverse effects of the pharmacological treatment of a number of targeted symptoms in 505 children with ASD. The authors found small to medium benefits to adverse effects ratios and concluded that individualized treatment is mandatory. Table 3 summarizes the medical indications and available drugs.

#### ADHD

ASD and ADHD share genetic, neurophysiological, and clinical similarities [10, 181]. Both disorders affect attention, flexibility, planning, and response inhibition, have a high heritability, early onset, overlapping comorbidities, and prevail in males [50, 58]. Hans Asperger already described attention problems as “almost regularly occurring in children of this type” [13]. Ronald et al. [265] found significant correlations between ASD and ADHD pheno and genotypes in their twins’ early development study, and a probability of 41% for co-occurrence ADHD in ASD patients. Nijmijer et al. [225] found genetic linkages between ASD and ADHD on chromosomes 7, 12, 15, 16, and 18. The “dual disorder” is characterized by increased psychopathology and psychosocial stress, more compromised cognitive and daily functions, including maladaptive behaviors, and poorer effects of therapy [48, 125, 147, 160, 246, 251]. ASD and ADHD share multiple comorbidities, such as dysexecutive problems, increased anxiety, sensory integration, sleep, affective and central hearing processing disorders, developmental delay, OCD, and epilepsy [187, 223, 281]. These comorbid conditions will largely determine the clinical picture. Unfortunately, ADHD in autistic patients is generally not appropriately treated [160]. This could be due to the fact that ADHD was excluded in autism diagnosis in ICD-10, a path that has now been changed in DSM‑5 and ICD-11.

Treatment of ADHD in patients with ASD should follow the same multimodal algorithms as for ADHD alone and should include psychoeducation [87, 219, 238], parental training [41, 85, 87], school-based measures (such as daily record cards [70, 80, 97], structured task organization, physical activity [39, 158, 302]), and medication [31, 285, 296]. ADHD medication is usually less effective, and SE are more pronounced in ASD patients, especially in those with ID [48, 85, 241, 255]. Cognitive training [56] and neurofeedback [88, 212, 252] are less effective and more complex. Occupational therapy [49] is useful as an adjunct for improving comorbid sensory integration and dysexecutive problems.

Medication for ASD/ADHD targets modulating dopamine and epinephrinergic transmitter systems, thereby increasing dopamine availability in frontal areas and striatum, and downregulating dopamine moderators. Usually, two types of medication are distinguished: stimulants (methylphenidate, amphetamine, lis-dexamphetamine) and nonstimulants (atomoxetine and alpha‑2 agonists).

*Stimulants.* Effectiveness and compatibility of methylphenidate, the most frequently used ADHD medication, have multiply been proven in patients with ASD and ADHD, with and without ID [11, 255, 282, 298]. In addition to the main ADHD symptoms, executive and nonexecutive memory, reaction time, reaction time variability, response inhibition, social communication, and self-regulation are significantly improved with methylphenidate [51, 149, 298] with somewhat lower effect sizes (around 0.5) in children with ASD and ADHD, compared to normally developing children with ADHD. Because of the short $t$ $1/2$ of about 2 hours, stimulants are usually administered in a slow-release formulation, acting for 10–14 hours, depending on the preparation. About 70% of the normally developing children and half of the children with ASD and ID respond by improved behavior, especially with decreased impulsivity, improved cooperation and attention, and less hyperactivity. Behavioral improvement is more pronounced in children presenting with hyperactivity and normal IQ [4]. Careful dosage titration is recommended because of the large variability of efficacy that may be explained genetically [206]. The effect of methylphenidate on growth has been divergently debated with height deficits ranging from 0 to 4.7 cm with consistent use [258]. In children with severe side effects or decreased responsiveness to methylphenidate, amphetamine [284], or lisdexamphetamine [52, 54, 127, 145], an inactive amphetamine precursor that is activated in the erythrocytes may be recommended because of their larger effect sizes. Amphetamines, and especially lisdexamphetamine, also improve mood while acting.

Emotional dysregulation (irritability) is a common problem in children with ADHD and with ASD, with rates around 78% for both disorders [179]. Stimulants and atomoxetine act effectively but may also increase emotional dysregulation, although at a much lower prevalence of about 17% [104]. In addition, effects on sleep (longer sleep latency, decreased sleep efficiency, and shorter sleep duration) were observed with stimulant medication [167].

*Atomoxetine.* The norepinephrine reuptake inhibitor and NMDA receptor antagonist possesses good effectiveness [123, 124] and (compared to methylphenidate) a considerably longer $t$ $1/2$ of 35 hours and 99% plasma albumin binding. Because of its nearly continuous action, atomoxetine is a recommendable alternative to methylphenidate, although with a smaller effect size [5, 236, 244], especially in children who respond with pronounced SE to stimulants or are very difficult to handle in the morning and evening hours, when methylphenidate does not act. It may also be recommended in children with comorbid depression, tics, or anxiety disorders [3, 5]. Atomoxetine needs a longer dosing period (up to 12 weeks) and may cause initial fatigue, headache, and gastrointestinal SE, wherefore the medication should initially be started in the evening hours. About 15% of the patients may react with increased aggression, requiring discontinuation of atomoxetine and either addition of risperidone [207] or aripiprazole [231] or switching to extended-release guanfacine [269, 270] or lisdexamphetamine [52].

Comparing atomoxetine and amphetamine derivates, higher effect sizes of methyplhenidate slow release preparations have been reported [121]. Small but significant cardiovascular effects have been reported for stimulant and atomoxetine medication [132], mainly small increases of the heart rate and of systolic or diastolic blood pressure [132]. Because significant cardiovascular effects may not be excluded in a small subgroup of patients (e.g., with slow drug metabolism), occasional blood pressure checks are recommended.

*Alpha-2-agonists.* Clonidine and extended-release guanfacine are less effective medications against ADHD core symptoms with some antitic potential, pronounced tiredness, and gastrointestinal SE, which may lead to discontinuing the medication. Hyperactivity and impulsivity are improved in about 45% of cases [144, 199, 241, 270, 294].

*Other treatments for ADHD.* Mindfulness-based [1, 259, 268] and neurofeedback therapies [138] have been tried with some success in children with ASD and ADHD.

#### Affective Disorders

Due to the fact that antidepressant medication is of questionable effect in children and adolescents with ASD, their use may generally not be recommended. There is no clear-cut evidence that this recommendation is also valid for patients with severe depression, and the widespread use of antidepressant medication reflects this challenge, especially in the light that the prevalence of comorbid depression in autistic patients is fourfold compared to the nonautistic population [318]. Combining antidepressants with (low-dose) antipsychotic medication may generally be recommended for augmenting antidepressant effects in therapy resistant depressive patients and–although with low evidence [78]–in suicidal patients. This relates to the long period needed for antidepressant drug effects to become evident and to the effect of antipsychotics to reduce initially present internal drive and suicidality. Psychotherapy adds to antidepressant therapy for light to medium severe depression in the short term but better in the long term. For severe depression, combining psycho and pharmacotherapy is recommended in normotypic children [40, 65].

Suicidality has been reported in 21.3% (7–47%) of patients with ASD [142, 324]. Suicidal ideation is very common in adolescents with ASD, especially in Asperger’s autists, and is largely related to their increased vulnerability to stress, anxiety, and depression, their inflexibility, and their proneness to become bullied or sexually abused [142].

Bipolar disorders are detected in 6–21% of adult ASD patients [307], and 30% of bipolar I patients meet the criteria for ASD [161]. Data for children and adolescents are still lacking. Therapeutic options include SGA, valproic acid, AD medication if severe depressive symptoms are present, and lithium. Lithium medication also improves social functioning in animals and adults [190]. Its use may be especially limited in children because of the narrow therapeutic range, its effect on thyroid function, the resulting need of a highly compliant and supportive environment, and the considerable and poorly tolerated emotional indifference created by the drug [208, 277].

#### Anxiety Disorders

About 40% of children with ASD present with various anxiety disorders, phobias including social phobia, general, and separation anxiety disorder, and OCD [323]. They also often react with symptoms of anxiety or even panic in reaction to changes in their environment. An early study [292] reported beneficial effects with low-dose AD medication against anxieties. Stachnik et al [290] reviewed the beneficial effect of neuroleptics for anxiety disorders in children with ASD. High doses of antidepressants may reduce OCD symptoms in normotypic children. Unfortunately, their effectiveness is not confirmed in children with ASD [169, 222, 253], possibly because of the background similarities of ASD and OCD [271].

In general, the treatment methods of choice for fears and OCD are parent training, play therapy, and cognitive behavioral therapy (CBT) [6, 60]. Antidepressants in higher dosages may be tried in individual patients as an adjunct to cognitive therapies. Because of the poor flexibility of patients with ASD, CBT may be very laborious in autistic children and adolescents.

#### Medication Against Sleep Disorders

Medication may be helpful in inducing and improving disturbed sleep but should be provided with caution: melatonin will improve sleep rhythm in 85% of the children with ASD even in those without disturbed melatonin circadian rhythm at a daily dosage of 1–6 mg given 30 minutes before bedtime [108, 267]. Advancing sleep onset will require a smaller dose of 0.2–0.5 mg given 3–5 h prior to the desired sleep time [32, 175]^9^.

Other sleep stimulating agents, like valerian, passion flower, and hops provide placebo support; benzodiazepines, zolpidem, and zaleplon act on GABA receptors, helping in inducing sleep but usually have a long $t$ $1/2$, decrease REM sleep phases, but lead to habituation, to losing sleep induction effects during prolonged use, and to promoting anxiety [234]. Sleep-inducing antidepressants like trazodone are commonly used. For contraindications (tricyclics, mirtazapine), see Sect. 3.2.

Restless legs syndrome [59, 280]^10^, another syndrome disturbing sleep and quality of life based on a genetic predisposition, dysregulation of iron metabolism, and the dopaminergic system, suggest considering iron deficiency as a cause of sleep disturbance [308].

Other sleep-stimulating agents, like valerian, passion flower, and hops, provide placebo support; benzodiazepines, zolpidem, and zaleplon act on GABA receptors, helping in inducing sleep but usually have a long $t$ $1/2$, decrease REM sleep phases, lead to habituation, may lose sleep induction effects and promote anxiety during prolonged use [234]. Sleep-inducing antidepressants like trazodone^11^ are commonly used. For contraindications (tricyclics, mirtazapine), see Sect. 3.2.

Benzodiazepines, especially those targeting $\text{GABA}_{A}$ receptor subtypes, may attenuate ASD symptoms [216]. The clinical significance of this effect is not known at present^12^.

#### Convulsions and Epilepsy

Epilepsy (more than one convulsion) occurs in about 5–46% of children with ASD, (compared to 1–2% in children not on the spectrum), depending on the clinical sample and the severity of ID [287]. Comorbid epilepsy adds to the impact of ASD on quality of life [303] because of a number of additional problems, such as cognitive, speech developmental, sleep, affective, medical, social, and behavioral issues [90, 118]. Phenotypes and causes are still insufficiently researched.

Mitochondrial respiratory chain defects have been detected as an important link between epilepsy and ASD [315]. In addition, three ASD associated syndromes with known genetic cause, tuberous sclerosis, Rett’s syndrome, and fragile X syndrome, are associated with epilepsy. Another group of disorders, epileptic encephalopathies, have been described in the context of brain dysfunction and increasing autistic symptomatology [74], affecting about 40% of children with convulsions in early childhood. These include early myoclonic encephalopathies, West, Dravet, Lennox Gastaud, and Landau–Kleffner syndromes, myoclonus epilepsy in nonprogressive encephalopathies, and continuous spike waves in slow-wave sleep (CSWS) [303]. Risk factors include epilepsies with known structural defects, bilateral frontal EEG changes, and persistent hypsarrhythmia [303].

#### Gastrointestinal Issues

Gastrointestinal distress related to constitutional, behavioral, and inflammatory causes is frequently observed in children with ASD and may be related to altered ASD severity [140]. Alterations of the intestinal microbiota, permeability, and functioning may, for example, alter intestinal serotonin metabolism and cause hyperserotoninemia, alter immune responses, and even brain functioning and behavior via the gut–brain axis [12, 193]. Attempts to influence these disturbances by diets (such as a gluten-free diet), probiotics, antibiotic or other “treatments” such as detoxification, would need careful prospective randomized clinical trials, precise diagnostics, and well-established clinical algorithms. At present, this clinical evidence is not available [240]

#### Irritability, Aggression, Disruptive, and Self-Injuring Behavior

Impulsive aggression and related disruptive behavior, as well as self-injuring behavior are frequently observed in ASD/ADHD and are the leading cause for school suspension, clinical referrals, and ward admissions [182]. Positive parenting [71], early intensive psychosocial and behavioral interventions [60, 76], specific multisystemic programs, such as multisystemic therapy [131] or the Fast Track program [25, 55], and psychosocial interventions such as T‑MAY [279] or TRAAY [276], and group sessions for social competence [101] lead to significant improvements of adaptive behavior. Recommendations for medical treatment include stimulants (in the case of comorbid ADHD) and nonstimulant medication, SGAs (cf. Sect. 3.1), antidepressant and mood stabilizing agents [48, 68, 75, 91, 116, 159]. In addition to pharmacotherapy, behavioral and social competence training, and parental counselling are strongly recommended.

#### Sleep Disorders

Independently of their intellectual capacity, up to $2/3$ of children with ASD suffer from sleep problems: delayed sleep onset, frequent night awakenings, reduced total sleep time, dys and parasomnias [26, 57, 63, 157, 175, 189, 197, 205, 256, 308, 317]. These problems often persist into adulthood. The causes range from poor sleep hygiene and inconsistent parental behavior [317], (self) regulatory problems and central excitatory/inhibitory imbalance, delayed sleep pattern maturation, a disturbed hypothalamic-pituitary-adrenal axis, and decreased and dysrhythmic melatonin secretion to decreased binding of melatonin to its transporter protein and melatonin receptor dysfunction [57, 141, 202]. Recently, slow-release melatonin^13^ was approved by the European Medicines Agency for the treatment of sleep disorders in children with ASD from the age of 2. In addition, anxiety [305], ADHD/ASD associated sleep and sensory integration problems [126] leading to increased external stimulation (or decreased stimulus filtering), and cerebral convulsions may disturb sleep and quality of life of affected children and, consequently, of the whole family. Therefore, sleep diagnostics and treatment are important for both children with ASD and their families [174, 308].

Restless legs syndrome [59, 280], another syndrome disturbing sleep and quality of life based on a genetic predisposition, dysregulation of iron metabolism, and the dopaminergic system, suggest considering iron deficiency as a cause of sleep disturbance [308].

Behavioral measures [30, 283, 314] like fixed bedtime routine, providing sleeping cues and a low stimulation evening routine, supporting self-soothing behavior, light therapy^14^ [84], avoiding daytime sleeping, etc., and sensory integration therapy [325] have proven to be helpful, although with little evidence [30].

#### Chronic Tic Disorders, Tourette Syndrome, and Stereotypies

Chronic tic disorders and motor stereotypies are common comorbid movement disorders in children and adolescents with ASD [249]. The prevalence of chronic tic disorder is about 6.5% [281], about 10 times higher than in normally developing children. It is characterized by involuntary movements or utterings that vary in onset and frequency, depending on daytime and seasonal variations and stress exposure. Treatment is necessary if severity and frequency exceed subjective or environmental tolerance. Effective treatment options [249] (besides relaxation, stress reduction, and bio or neurofeedback) include antipsychotics such as risperidone, aripiprazole, or pimozide, eventually with added pentoxyfylline, and the anticonvulsant topiramate are effective, whereas haloperidole, levetiracetam, guanfacine, and atomoxetine, as well as metoclopramide and odansetron, have not proven effective [249, 262].

### Other Substances, Supplementary and Alternative Therapies

Among the “newer” pharmacologic concepts (such as IGF‑1, memantine, D‑cycloserine, arbaclofen, and oxytocin [240, 300]), only three show promise for the future: oxytocin with the objective to improve sociogenic behavior, beta blockers to reduce stress, and the glutamate antagonist, 2‑methyl-6-(phenylethynyl)pyridine (MPEP), to reduce stereotypic behavior [94]. For the latter substance, it is feared that sociogenic behavior may deteriorate during treatment [297].

In the short term, intranasal oxytocin enhances motivation and attention to social stimuli, improves social initiative, understanding, learning [8, 22, 176], and better recognition of emotions [111]. Unfortunately, these improvements were not substantiated in long-term trials [7, 112, 313, 321, 322]. A meta-analysis [248] reported medium-effect sizes for prolonged oxytocin therapy in small samples. Reasons for the variation in oxytocin response include time dependency of the oxytocin response [230], single nucleotide polymorphisms of the oxytocin receptor [148], and lasting effects of postnatal stimulation of the oxytocin system [300]. When studying oxytocin effects patients and targets must be carefully selected. Therefore, the clinical usefulness of oxytocin is still a matter of debate [228, 306]. Melanocortin, stimulating oxytocin release, could be a useful alternative [215], but large clinical trials are lacking. Still, a special edition of “Brain Research”^15^ provides a comprehensive overview about the state of research.

There is only limited evidence for using beta blockers for reducing stress-related autoaggressive behavior [312] or memantine for improving language and memory functions [233]. Defects of GABA-A receptors, leading to deficient synaptogenesis, have been demonstrated in fragile X syndrome, a pervasive developmental disorder with known genetic defect^16^. Ganaxolone, a strong GABA-A agonist, was used in a controlled clinical study [29, 188] and was found to be safe but only effective in a subgroup of patients with fragile X syndrome, high levels of anxiety, and low intellectual capacity.

Medical cannabis, especially for ADHD, tics, sleep problems, behavioral problems, and anxiety [2, 134, 247], may improve symptoms but does not lead to remission. Treatment evidence at present is limited to anecdotical reports and a few small studies; three further studies are to be expected. Treatment options should, therefore, be restricted to single patients in whom standard treatment did not improve severe symptoms.

Various behavioral and functional therapies, such as structured behavioral therapies [178, 254, 299], communication and social skills training [177, 213], occupational therapy [49, 194], mindfulness [259], play teaching [162], music [217, 289], and speech therapy, have been shown to have beneficial effects in improving development, behavior, speech, social functioning, and quality of life [146, 191, 192, 220, 221, 275]. Physical exercise is an effective treatment option, especially in children with dual disorder, ASD and ADHD [128, 286, 302].

Alternative, “natural” treatments seem less invasive, safer (there are no reports on dangerous action), more intuitive to understand, and easier to procure. Parents are concerned with the safety or side effects (listed in the package leaflet) of medication or are disappointed because conventional medication did not change the core symptoms of ASD [120]. Therefore, alternative therapies are very popular [186, 191, 316]; a third of the parents of children with ASD have tried “alternative”, “integrative”, or “complementary”^17^ therapies [185, 186, 191]. A higher educational level of the mothers predicted the use of alternative therapies [120]. Half of the families use alternative therapies, although they do not rate them as useful.

Most of these therapies are used as an adjunct to conventional therapy. Biologically based therapies (such as diet[239, 293], vitamins and minerals, food supplements such as omega‑3 fatty acids [150], herbal remedies, secretin), and mind–body interventions (such as prayer, shamanism, biofeedback, meditation, and relaxation) are more often perceived efficacious than body-based methods (such as sensory integration therapy [325], massage, craniosacral therapy, neurofeedback, and special exercises) or energy therapies (healing touch, energy transfer) [120]. Technology based interventions seem promising because of the attention sustaining potential, but, at present, evidence of the success of such approaches is poor [172, 250]. Examples are interventions for acquiring language skills [226], for differentiating facial expressions [19], treating food selectivity [20], or anxiety or stress management [37].

A number of physicians encourage multivitamins (49%), essential fatty acids (25%), melatonin (25%), and probiotics (19%), and discourage withholding (76%) or delaying immunizations (55%), chelation (61%), anti-infectives (57%), or secretin (43%) [120]. It has to be stated that there is no clinical evidence for applying specific (e.g., gluten-free or pro-biotic) diets [203], vitamins^18^ [155, 237], oligominerals, herbal medicine [311], transfer of energy, chelates^19^ [151], or biologicals such as secretin [180, 186]. It has been found that 10% of parents even use potentially dangerous “medication” such as “whole-brain extracts” [185]. Medication from the Far East, such as traditional Chinese medicine or acupuncture, or osteopathy may be useful in the short-term run in improving single symptoms (restlessness, sleep disturbance); the long-term outcome is rather dubious [45].

## Discussion

Pharmacotherapy in children and adolescents with ASD may be helpful in overcoming otherwise not resolvable behavioral and attentional problems (see Table 2 for an overview of indications and classes of useful substances). Individualized treatment is always mandatory, Reviewing the extensive literature on pharmacotherapy of ASD, a few trends may be recognized:

1. Conventional therapy, although mostly funded on extensive controlled studies, has its limits, especially when treating irritability and temper tantrums. These problems should be restricted by early behavioral treatment. Unfortunately, these treatments are tedious and not available everywhere. In addition, the question of the impact of comorbid conditions has not been solved as yet.
2. Pharmacologic treatments are not sufficient; the primary ASD treatment, especially for children with intellectual disabilities, will remain structured and functional therapy, as well as parental empowerment and support.
3. Therapies aiming at improving the core symptoms of ASD, such as social communication: novel therapies, e.g., oxytocin, are encumbered with the complex functioning of our social brain, which is outlined in the first days of life or even before.
4. At present, genetically based therapies are not visible on the horizon, mostly because the genetic background of ASD is so complex that it will probably need further years of intensive research to link clinical pictures to genetic variants and establish repair options.

Behavioral problems, including irritability, reactive and proactive aggression, disruptive and self-stimulating behavior, restlessness, and temper tantrums, are among the most important therapeutic targets in children with ASD. Because of their very limited flexibility [102] and working memory problems [117], children with ASD easily become despaired and helpless and express this in externalizing behavior that can become difficult to control. Pharmacologic treatment, mostly using antipsychotics, must find a compromise between behavioral control, oversedation, and (mostly metabolic) side effects.

Depressed mood and anxiety disorders call for psychotherapy and, in selected patients, for treatment with antidepressants. The problems with antidepressant medication are its reduced efficacy in autistic vs. normally developing children (see Sect. 3.2), and, again, walking the tightrope between brightening mood or reducing anxiety or obsessions and compulsions and an increased behavioral activation.

Sleep problems are observed in a majority of patients with ASD. Sleep hygiene and bedtime routines should be tried before trying medication, and sleep-related side effects of stimulant therapy should also be considered as a promoting factor of sleep dysfunction. Melatonin is the first-line drug, especially for difficulties in falling asleep. It is effective in about two-thirds and counterbalances inherited melatonin dysfunction. It should be noted that falling asleep with lights on (especially from computer or mobile phone screens) counteracts the action of melatonin medication.

Treatment of ADHD, one of the most prominent comorbid conditions of ASD with overlapping symptoms, is often a key factor in enabling social and intellectual learning, school attendance, and fighting restlessness and impulsivity. Problems are related to the reduced efficacy of pharmacotherapy compared to normotypic patients and a multitude of interacting problems, e.g., bipolar disorder and ADHD.

Convulsions, most frequently observed in children with ASD and ID, should be treated like in normally developing children (see Sect. 3.2.1). Attention should be paid to sedation, metabolic, learning inhibition side effects, and, and in adolescents, to teratogenic side effects for the offspring.

The rediscovery of the gut–brain axis is a relatively new field of research and might, therefore, be overestimated by parents. More prospective studies will shed light on the effects of dietary and probiotic measures. Alternative treatments are comprehensively largely overestimated for their effects, ranging from dietary to physical and possibly endangering measures. Because alternative “medications” are not controlled for their action in prospective randomized trials, it is difficult to argue against the use of such substances in the general public, mostly because “natural” substances are considered harmless and innocuous (see Sect. 3.4).

In summary, we compiled an overview on substances that may be advantageously used in children with ASD with the aim of improving social behavior, learning ability, and quality of life of the children and their environment. The approach is rather defensive, mostly targeting undesired symptoms. Future work and experience should focus on desired changes of core symptoms, on long-term efficacy, on reducing polypragmasia and undesired drug effects, and on avoiding overtreatment, especially if behavioral therapies are available as an alternative. On the other hand, the benefits of carefully prescribed medication should always be recognized.

## Footnotes

1 applied behavioral analysis [209].

2 Treatment and Education of Autistic and related Communication Handicapped Children [211].

3 Picture Exchange Communication System [89].

4 N‑methyl-D-aspartate.

5 Again, selecting more carefully performed studies, more recent, often cited papers, and preferring reviews, if available, over original studies.

6 norepinephrine and dopamine reuptake inhibitor.

7 With inconsistent results [129, 137].

8 Electrical status epilepticus during slow-wave sleep.

9 These two references do not primarily refer to children with ASD.

10 General description.

11 Trittico®.

12 Alterations of the excitatory/inhibitory CNS imbalance in children with ASD? [99].

13 Slenyto®.

14 10.000 lux for $1/2$ h in the early evening and/or morning in order to synchronize the circadian rhythm better.

15 Vol. 1580:1–232(2015).

16 Fragile X mental retardation 1 (FMR1) gene on chromosome X (Xa27.3).

17 Alternative and conventional medication.

18 This is disputed for vitamin D: evidence [155] vs. no evidence [204].

19 For heavy metal detoxication.
